# Blood Demand and Challenges for Patients With Beta-Thalassemia Major in Eastern Saudi Arabia

**DOI:** 10.7759/cureus.17470

**Published:** 2021-08-26

**Authors:** Muneer H Albagshi, Mona Saad, Abdulmohsin M Aljassem, Abdulaziz A Bushehab, Noura H Ahmed, Mahmoud M Alabbad, Nawal Omer, Osama A Alhamad, Tarig A Sultan, Samy Bahgat

**Affiliations:** 1 Pediatric Hematology, Hereditary Blood Diseases Center, Al Ahsa, SAU; 2 Internal Medicine/Hematology, Hereditary Blood Diseases Center, Al Ahsa, SAU; 3 Internal Medicine, Hereditary Blood Diseases Center, Al Ahsa, SAU; 4 Nursing, Hereditary Blood Diseases Center, Al Ahsa, SAU; 5 Pediatrics, Hereditary Blood Diseases Center, Al Ahsa, SAU; 6 Medical Affairs, Hereditary Blood Diseases Center, Al Ahsa, SAU; 7 Integrative Medicine, Hereditary Blood Diseases Center, Al Ahsa, SAU; 8 Blood Bank, Maternity and Children Hospital, Al Ahsa, SAU

**Keywords:** beta-thalassemia major, packed red blood cell transfusion, hypertransfusion, saudi arabia, hemoglobin disorders

## Abstract

Background

β-thalassemia major is a hereditary disorder of hemoglobin (Hb) that results in defective Hb synthesis, leading to severe chronic anemia. The mainstay of its treatment is lifelong regular packed red cell transfusions associated with iron-chelating therapy. Globally, there is a gap between blood donation and the actual needs of the patients who depend on transfusion. Patients with β-thalassemia major are no exception and have limited access to regular and safe blood transfusions. This study aimed to assess the gap between the demand and supply of blood for transfusion-dependent patients with β-thalassemia major treated at the Hereditary Blood Diseases Center, Al Ahsa, Eastern Saudi Arabia.

Methodology

This was a retrospective, cross-sectional study conducted at the Hereditary Blood Disease Center, Al Ahsa, Saudi Arabia, including patient data from January 2017 to December 2019. We used Excel 365 from Microsoft Office 2016, version 1706.

Results

A total of 158 patients were on chronic transfusion. Of the total patients, 65% were adults, while the remaining 35% comprised the pediatric population. The total number of units requested and received during the three-year period was 14,509 and 9,530, respectively, indicating a gap of 4,979 (34%) units. The age of most of the units received was more than 14 days: 36% of those in 2017, 49.9% in 2018, and 61.5% in 2019. Rare blood groups and alloimmunization accounted for <8% of the patients. Prestorage filtration was the policy for all units.

Conclusions

There was a gap between the demand and supply of blood for patients with β-thalassemia major treated at our center. We suggest raising awareness regarding the high demands for fresh red blood cell components in patients with thalassemia major, encouraging voluntary blood donations, enhancing national blood-banking policies, and reducing the fragmentation of blood services to reduce the gap between demand and supply.

## Introduction

Thalassemia syndromes are a heterogeneous group of disorders that include a wide spectrum of clinical manifestations and genetic disturbances that involve α and β gene defects leading to α- and β-thalassemia, respectively. These genetic defects lead to reduced hemoglobin (Hb) synthesis [1,2], consequential hemolysis, and reduced survival of red blood cells resulting in chronic anemia. In severe phenotypes, bone marrow expansion occurs [3]. Both α- and β-thalassemia are autosomal recessive disorders [4,5]. Globally, thalassemia is the most common hereditary disorder and is endemic in the Mediterranean region, the Middle East, the Indian subcontinent, the Far East, and tropical Africa. Currently, in many nonendemic areas, thalassemia is reported at a relatively high frequency [6].

Depending on the clinical features, β-thalassemia is grouped into the following three categories: β-thalassemia carrier state, thalassemia intermedia, and thalassemia major [5,6]. Without effective therapy, β-thalassemia major is fatal. Regular red blood cell transfusion along with iron chelation therapy is the standard of care, while bone marrow transplantation is the only cure if a suitable stem cell donor is available. The life expectancy of patients may approach normal with either of the therapeutic options [7]. Globally, the annual number of donated units of blood is approximately 120 million units; however, there is still a shortage of supply against demand, and many patients do not have timely access to safe blood, especially in low-income countries [8].

In this study, we aimed to assess blood supply against blood demand for patients with β-thalassemia major receiving regular blood transfusion therapy at the Hereditary Blood Diseases Center at Al Ahsa, Eastern Province, Saudi Arabia.

## Materials and methods

This retrospective, single-center, cross-sectional study was conducted at the Hereditary Blood Diseases Center, Al Ahsa, Saudi Arabia. In this study, we included demographic data and transfusion details of patients from January 2017 to December 2019. We used Excel 365 from Microsoft Office 2016, version 1706, for descriptive statistics and creating figures and tables. We included all patients with β-thalassemia major receiving regular blood transfusion therapy at the center from January 2017 to December 2019. Data were retrieved from patient’s records.

## Results

**Figure 1:**
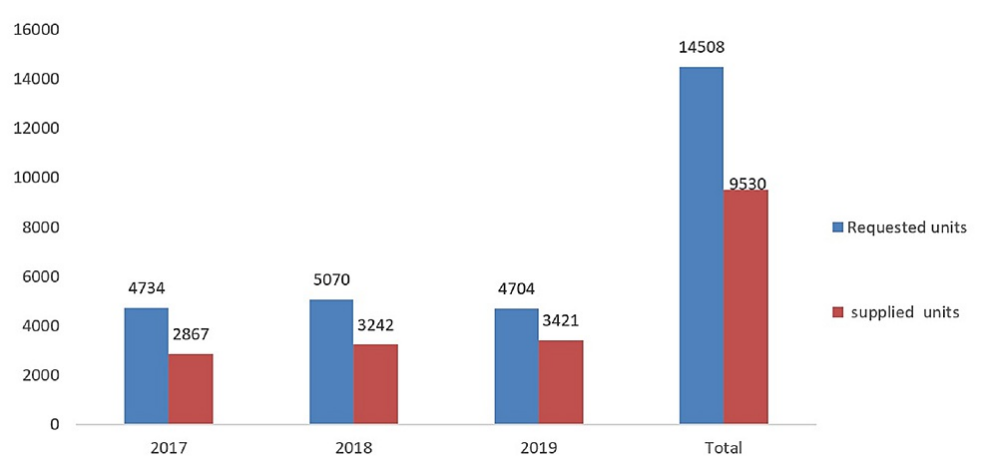
Annual requested and received red cell units over the three-year period.

**Figure 2:**
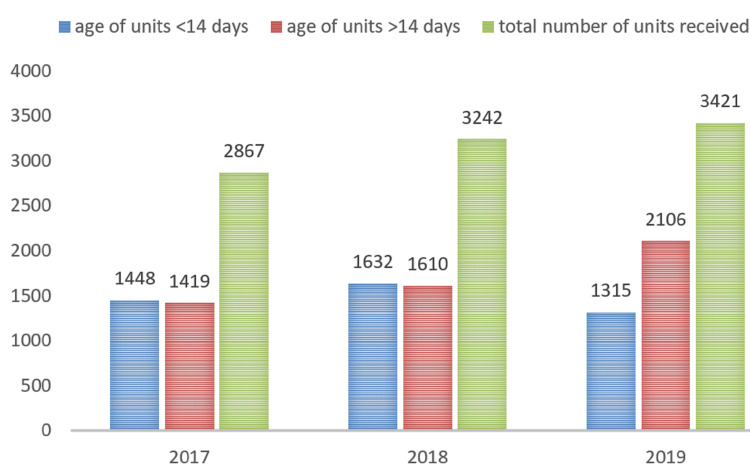
Age of the transfused red cell units.

A total of 158 patients were under chronic transfusion therapy aged five months to 54 years with a mean of 34 years. Most patients were native Saudis (Table 1). The mean pretransfusion Hb was 8.9 g/dL + 1.4 g/dL (standard deviation). The average monthly red blood cell unit consumption was 411 (Table 2). The supply deficiency for the three-year period was 39.4% in 2017, 36.1% in 2018, and 27.3% in 2019. Blood supply against blood demand during the study period is shown in Figure 1. There was an improvement in blood supply each year, albeit insignificant. The blood supply affected adult patients the most as they accounted for 75% of the deficient units compared to the pediatric population that accounted for 25% of the total deficient units. The age of transfused blood units increased with time, especially in 2019, as shown in Figure 2.

**Table 1:** Characteristics of β-thalassemia major patients receiving chronic red cell transfusion.

Parameter	Number of patients	Total
Age	>14 years: 103 (65.2%)	158 (100%)
	<14 years: 55 (34.8%)	
Sex	Male: 85 (53.8%)	158 (100%)
	Female: 73 (46.2%)	
Nationality	Saudi: 154 (97.5%)	158 (100%)
	Non-Saudi: 04 (2.5%)	

**Table 2:** Packed red cell transfusion units required monthly.

Packed red blood cell units per order	Number of units requested
Four units	120
Three units	225
Two units	60
One unit	6
Total units per month	411

## Discussion

The mainstay in the management of transfusion-dependent β-thalassemia (TDT) is regular lifelong packed red cell transfusions and iron chelation [9]. Without treatment, these patients usually die in the first or second decade of life [10-12]. Red blood transfusions correct chronic anemia, facilitate normal growth, suppress ineffective erythropoiesis, prevent bone deformities, and reverse hepatosplenomegaly. Regular transfusion of fresh red blood cells normalizes the patient’s activity levels, which, in turn, improves their quality of life [11,12]; however, chronic red blood cell transfusion is not free of adverse events. Such complications can be rapid in onset such as acute hemolytic reactions, severe allergic reactions, and septic shock, while delayed complications include transfusion-transmitted infections, delayed hemolytic transfusion reactions, and alloimmunizations. Iron overload is the major cause of morbidity and mortality [10,13] and is clinically reflected by endocrine disorders, such as growth retardation, failure of sexual maturation, diabetes mellitus, and insufficiency of the parathyroid, thyroid, and pituitary hormones, along with dilated cardiomyopathy, liver fibrosis, and cirrhosis [14]. When started early in therapy, iron chelation prevents or minimizes complications related to iron overload in patient’s organs and enhances their quality of life and life span [11,15]. The cost of TDT management is high, and in many countries, it is the government’s responsibility as they manage blood donor recruitment and blood management in blood banks [16,17]. Thalassemia centers were created to unify management programs, reduce the cost and fragmentation of care, and, consequently, provide adequate monitoring, good adherence to treatment, patient support, and counseling [17,18].

Our study of 158 patients (Table 1) revealed that, for the three-year period, 14,508 units of packed red cell units were ordered and 9,530 units were received (Figure 1), that is, 34% of demanded units were not supplied. The blood supply shortage worsens the predicament of patients with thalassemia. In a report from the World Health Organization [19], of the patients who needed a transfusion, only 3% received it in some parts of Africa. In America, almost half of the patients received less than adequate transfusion, and, globally, as low as 12% of children born with TDT were transfused [20,21].

Almost 50% of units supplied were aged 14 days or more (Figure 2) which reflects the paucity of donation and availability of fresh blood components, especially as noted in 2019. Red cells are stored in bags at 2-8°C for up to 42 days, depending on the red cell preservation solution used. These stored red blood cells progressively undergo a series of metabolic and rheological changes referred to as the storage lesion. These lesions include biomechanical and immunologic changes that affect red cell viability, deformability, oxygen-carrying capacity, microcirculatory flow, and recipient response [22-24]; however, some but not all of these changes are reversible in vivo after transfusion [25-28]. Researchers have proven the superiority of fresh red blood cells in both improving the outcome and reducing mortality [28,29], and the quality of evidence is currently to transfuse red cell units within 14 days or less, which reinforces the importance of providing more blood supply to thalassemia centers [28]. The fresh blood supply prolongs the time interval between transfusions and, in turn, reduces the total number of consumed units, service costs, and iron overload [2].

This study demonstrates a significant shortage of blood supply against the demand required for patients with TDT. Factors that contribute to the reduction of blood donation and have a negative impact on the supply include a lack of national authority, adequate financing, quality systems, information technology support, hemovigilance policies, fragmented and inefficient services, varying quality standards, and a lack of infrastructure and political support [29,30].

## Conclusions

We recommend raising the awareness of β-thalassemia on social media, encouraging voluntary blood donations, increasing donation centers, and continuous blood donation campaigns. The centralization of blood banks in the provinces is an excellent option to enhance the national blood-banking policies, reduce the fragmentation of blood services, and narrow the gap between demand and supply.
